# Integrating local and scientific knowledge: The need for decolonising knowledge for conservation and natural resource management

**DOI:** 10.1016/j.heliyon.2023.e21785

**Published:** 2023-11-02

**Authors:** Malaika P. Yanou, Mirjam A.F. Ros-Tonen, James Reed, Kaala Moombe, Terry Sunderland

**Affiliations:** a Amsterdam Institute for Social Science Research (AISSR), University of Amsterdam, Amsterdam, the Netherlands; b Center for International Forestry Research, Bogor, Indonesia; c School of International Development, University of East Anglia, Norwich Research Park, UK; d University of British Columbia, Vancouver, Canada

**Keywords:** Knowledge integration, Knowledge co-production, Indigenous and local knowledge, Decolonising knowledge, Politics of knowledge, Conservation, Natural resource management, Southern Africa

## Abstract

Integrating Indigenous and local knowledge in conservation and natural resource management (NRM) initiatives is necessary to achieve sustainability, equity, and responsiveness to local realities and needs. Knowledge integration is the starting point for converging different knowledge systems and enabling knowledge co-production. This process is also a key prerequisite towards decolonising the research process. However, power imbalances may perpetuate dominant forms of knowledge over others, obstruct knowledge integration, and eventually cause the loss of knowledge of the marginal and less powerful knowledge holders. Despite increasing interest in knowledge integration for conservation, NRM, and landscape governance, documentation of integration processes remains fragmented and somewhat scarce. This semi-systematic literature review contributes to filling this gap by synthesising methods, procedures, opportunities, and challenges regarding integrating and decolonising knowledge for conservation and NRM in Southern Africa. The findings demonstrate that despite an increasing number of studies seeking to integrate Indigenous and local knowledge and scientific knowledge relevant to conservation and NRM, methods, procedures, and opportunities are poorly and vaguely documented, and challenges and colonial legacies are often overlooked. Documentation, valuing Indigenous and local knowledge, addressing power relations, and collaboration across knowledge systems are missing steps towards efficient knowledge integration. The paper concludes that there is a need for further research and relevant policies. These should address methods and implications for equitable knowledge integration processes and move beyond knowledge sharing and mutual learning towards decolonising knowledge for conservation and NRM.

## 1 Introduction

Indigenous and local communities have a long history of land tenure and natural resource management (NRM) and have developed and adapted knowledge and resource-use practices that help navigate complexity [1]. As such, there has been an increasing acknowledgement of the importance of Indigenous and local knowledge (ILK)^1^ for maintaining biodiversity in global science-policy negotiations since the Brundtland report by the World Committee on Environment and Development [2]. These include the Convention on Biological Diversity (CBD) adopted at the 1992 Rio Earth Summit [3], the report of the Intergovernmental Platform on Biodiversity and Ecosystem Services (IPBES) [4] and the post-2020 Global Biodiversity Framework [5,6]. These and other initiatives, reports and agreements highlight the significance of ILK for local adaptation to global environmental change, biodiversity and forest conservation, along with sustainable land and NRM [[7], [8], [9]].

In academia, knowledge integration and other co*-*strategies where all participants validate both the diverse meanings and contents of knowledge systems and the co-produced outcomes are acknowledged as paving the way towards knowledge legitimacy and applicability [10,11]. A growing scholarship seeks to engage and cooperate with ILK holders in whose territories they conduct research [[12], [13], [14]]. When this occurs, different knowledge holders begin to interact. When the data collection process is not extractive, a “project of integration” – as Nadasdy [15] called it – might be taking place, particularly between ILK and scientific knowledge holders. However, such processes often imply that ILK conforms to scientific knowledge, and knowledge integration appears to be a mere technical exercise to incorporate the ILK of a minority group into a majority system. In doing so, they may fail to consider the political dimensions of the issues of the process and inadequately address inequalities and power relations [[15], [16], [17]].

On the contrary, integration should be considered a process that establishes equitable collaboration amongst different knowledge holders by empowering the most marginalised knowledge holders [[18], [19], [20], [21], [22]]. In this regard, the relevance of knowledge integration becomes threefold: maintaining biocultural diversity, filling gaps in scientific know-how, and recognising ILK as fundamental to social justice, sovereignty, autonomy and identity of Indigenous peoples and local communities (IPLCs) [23].

However, the dilemma is that the dominant conservation policies are still driven by actors from the Global North and rooted in colonial constructs such as protected areas and national parks [9,13,24]. Thus, in certain settings, colonial conservation narratives continue to prevail in national policies. Such colonial conservation models have largely ignored the knowledge and practices of IPLCs who inhabit, rely on, and often sustain their ancestral lands. Effectively engaging with ILK systems involves encountering different world views, identities, practices, and ethics in a context of asymmetries of power and rights [4,7]. At the science and policy level, the challenge is to move towards new ways of doing and knowing, overcoming the limits of a single knowledge system and better supporting endogenous development [[25], [26], [27], [28]].

While there is growing literature on knowledge integration, knowledge governance, and co-production in environmental governance [19,27,[29], [30], [31], [32]], few studies show how this occurs in practice. Against this background, this paper assesses how the knowledge integration rhetoric is realised in conservation and NRM research and initiatives. Based on a semi-systematic literature review methodology outlined in the next section, this paper reviews peer-reviewed studies on knowledge integration in environmental and conservation research and other initiatives in Southern Africa.^2^ We particularly focus on the procedures and methods used to promote knowledge integration and the power and ethical challenges these projects face. More specifically, this paper aims to delve deeper into how ILK is integrated into environmental and development projects and practices and what opportunities and challenges are encountered in this regard. In doing so, the paper aims to contribute insights into decolonising knowledge efforts^3^ and examine how to operationalise such processes.

The paper specifically addresses the following review questions:

1. What knowledge integration projects can be identified in the conservation and NRM research field in Southern Africa?
2. What kinds of knowledge do the studies aim to integrate?
3. What methods and procedures do conservation and NRM projects and initiatives apply to integrate scientific and ILK?
4. How can the methods be classified in terms of the inclusivity of participation and ILK?
5. What are the opportunities and challenges of knowledge integration and co-production in these initiatives?
6. To what extent does the debate on decolonising knowledge play a role in efforts towards knowledge integration and co-production?

The rest of this paper is structured as follows. The next section presents the methods used for this review. After presenting the evidence base and answers to the review questions, we discuss the methods, opportunities and challenges, and the politics and decolonisation of knowledge integration. In the concluding section, we present recommendations for a future research agenda that addresses the gaps identified in this review.

## 2 Materials and methods

### 2.1 Search strategy

We ran a three-step approach to identify and select relevant information and data. We first searched for papers using Scopus, Web of Science, and Google Scholar to identify relevant case studies in seven Southern African countries – Zambia, Zimbabwe, Namibia, Botswana, Swaziland, South Africa, and Lesotho – that describe and explain different stages in knowledge integration processes.

Firstly, we tested several search strings, including the following (“knowledge integration” OR “knowledge co-production”) AND “scientific knowledge” that did not yield relevant results in Scopus and Web of Science. We then broadened the search string to (“traditional ecological knowledge” OR “Indigenous knowledge” OR “local knowledge”) AND (conservation OR “natural resource management”) AND (Zambia OR “South Africa” OR Zimbabwe OR Namibia OR Botswana OR Swaziland OR Lesotho),^4^ which resulted in 823 papers (719 after duplicates removed) for the period 1992–2021. We took the 1992 Rio conference as a pivotal date representing a watershed moment due to adopting a set of guiding principles on environment and development (Rio Declaration). Moreover, the Conference was a historical event that largely launched a new way of thinking about the linkage between development and environmental processes [33].

**Fig. 1:**
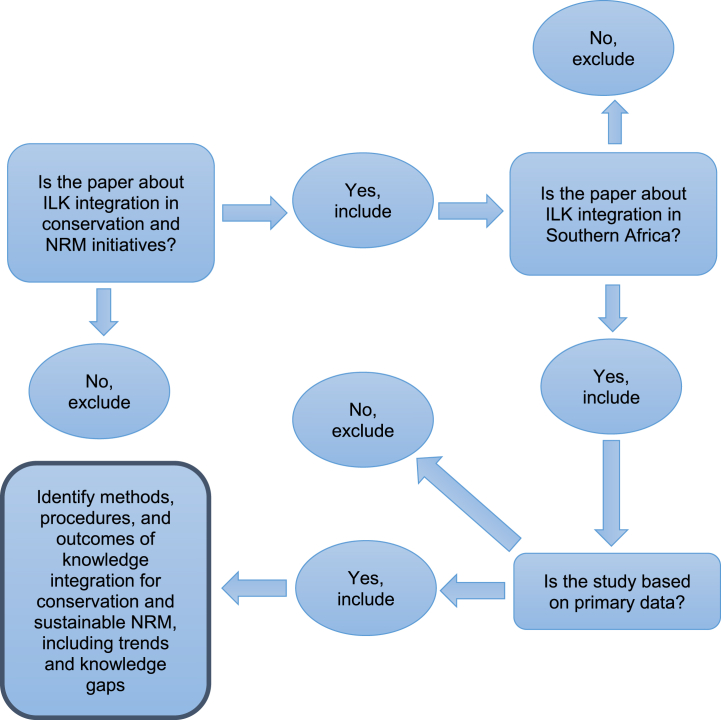
Decision tree (start from the top left corner and proceed clockwise) used to select literature (Adapted from Ref. [26]).

Next, we hand-searched the tables of contents of the last five years of four relevant journals^5^ (using the above search strings) and, subsequently, the reference lists of key papers on the topic. This generated seven additional relevant papers. Finally, we used a decision tree based on inclusion and exclusion criteria to select documents for the review (Fig. 1). Grey literature (such as project documents and reports) was excluded due to resource constraints and concerns about comparability and quality. We are aware that such a decision implies a risk of excluding relevant empirical material.

We want to highlight two points regarding the terminology used in this review. First, we acknowledge the complexities and sensitivities associated with certain terminology. For example, knowledge integration can be co-opted and rather reflect assimilation. However, to capture a sufficient breadth of literature, we used the search term knowledge integration as a broader and more generally used term than knowledge co-production, co-creation or – more recently – knowledge weaving [[34], [35], [36]] and knowledge braiding [[37], [38], [39]]. Second, we use the definition of ILK widely used by international organisations such as UNESCO, IPCC, and the FAO (see footnote 1) [40, p. 5]. We use that term consistently unless we cite a paper that explicitly uses another term.

### 2.2 Inclusion criteria

**Fig. 2:**
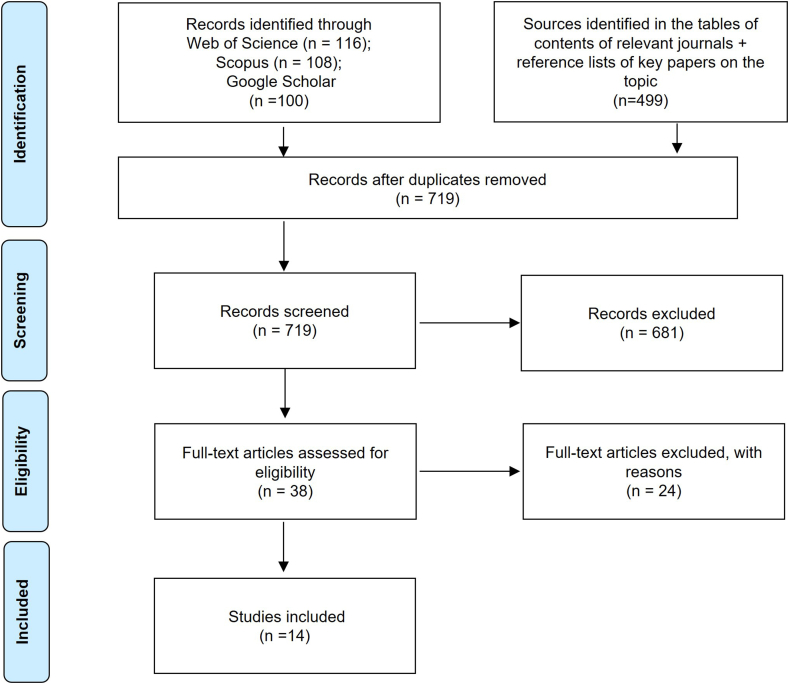
PRISMA flow diagram showing the document sources, screening process, and output of the literature selection (Adapted from Ref. [40]).

We used a decision tree based on the inclusion and exclusion criteria in Table 1 to identify relevant documents for the review [1] (Fig. 1). We then proceeded with title and abstract screening, after which 38 papers remained for full-text screening. After screening these full texts, 14 papers were included in the review (Fig. 2; Table 2).

**Table 1:** Inclusion and exclusion criteria for the review based on the population-intervention-comparator-outcome (PICO) framework.

Criteria	Inclusion	Exclusion
Population	Research conducted in the Southern Africa region (Zambia, Zimbabwe, Namibia, Botswana, South Africa, Swaziland, Lesotho).	Studies that fall outside of the geographic scope of the region.
Intervention	Case studies in which scholars or practitioners initiated a project, NRM or conservation initiative aiming at knowledge integration.	Theoretical studies and studies not related to knowledge integration relevant for conservation and NRM, such as weather prediction, climate change adaptation strategies, medicinal plants, etc.).
Comparator	Case studies that compare methods and approaches for conservation and NRM knowledge integration strategies 1) in different geographical settings and 2) through time.	
Outcome	Results demonstrate how and why knowledge integration strategies and methods work or do not work efficiently towards more effective conservation and NRM.	The studies do not show empirical results on knowledge integration strategies and methods.
Additional criteria	Articles in English, French, and Portuguese. Based on empirical data.	Articles in other languages, synthesis studies and studies based on literature review.

**Table 2:** Variables used in the analysis.

Review question	Variable	Operationalisation
1. What knowledge integration projects can be identified in Southern Africa in the field of conservation and NRM?	Location	Identify conservation and NRM knowledge integration projects in Southern Africa.
	Scope of the initiative Period of implementation	Identify the final aim of the integration process (a) creating new knowledge (searching for new solutions); b) Empowering local voices (creating meaningful solutions for local actors); c) Power relations (address power unbalances amongst different actors; d) influence policy (provide relevant information for evidence-based policymaking). Years during which the initiative was in place.
2. What kinds of knowledge do the studies aim to integrate?	Knowledge systems	Types of knowledge that are being integrated (experts, practitioners' knowledge, ILK).
3. What methods and procedures do conservation and NRM projects and initiatives apply to integrate scientific and ILK?	Methods	Procedures followed to achieve knowledge integration.
4. How can the methods be classified in terms of the inclusivity of participation of ILK holders?	Degrees of collaboration	Identify the level of collaboration between different knowledges and knowledge holders (Fig. 3) and the kind of scientific and ILK integrated.
5. What are the opportunities and challenges of knowledge integration and co-production in these projects?	Opportunities	The reviewed articles identify positive outcomes and opportunities for knowledge integration in conservation and NRM.
	Challenges	The reviewed articles identify challenges/problems regarding knowledge integration in conservation and NRM.
6. To what extent does the debate on decolonising knowledge play a role in efforts towards knowledge integration and co-production?	Stewardship Decolonial issues Authorship	Who leads the process? Capture if and how initiatives address (or not) different decolonial perspectives. Capture the role of local and external authors in the publication.

### 2.3 Data analysis

**Fig. 3:**
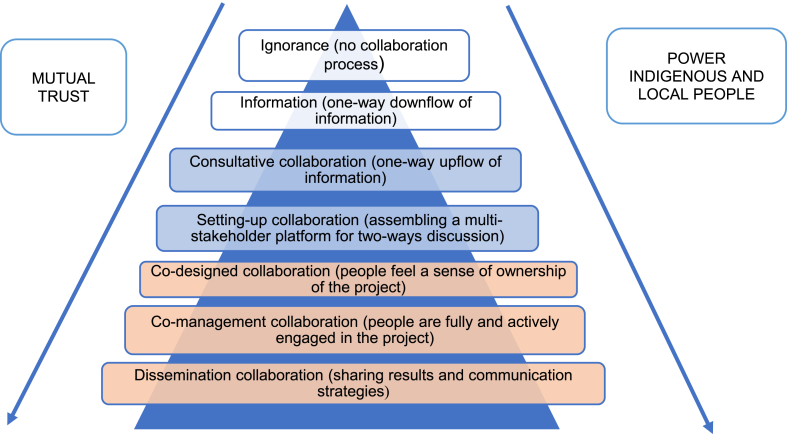
Degrees of collaboration amongst different actors involved in the research (e.g. researchers, local communities, practitioners). We divided the seven steps into three groups based on their level of collaboration. Ignorance and information steps represent low collaboration; consultative and setting-up collaboration steps are medium collaborative; and co-design, co-management, and dissemination steps represent a high degree of collaboration (Adapted from Ref. [42]).

This review follows an exploratory and descriptive design, using a narrative synthesis of knowledge integration methods, procedures, and outcomes [41]. More specifically, we extracted data using six variables (Table 2), partly based on our review questions, and partly adapted from Ref. [42]. For the first review question on knowledge integration projects in conservation and NRM, we extracted data on the projects’ implementation period, location within Southern Africa, and scope. For the second and third review question on methods and integration of the procedures, we extracted information on the approaches towards knowledge integration and assessed the inclusively of these methods based on the degree of collaboration to identify the levels of interactions amongst ILK and scientific knowledge systems. Drawing from Ref. [42], we classify degrees of collaboration on a continuum ranging from ignorance to co-designed research (Fig. 3). We focused on positive and negative perceptions of the knowledge integration process for the question on opportunities and challenges. Finally, analogous to the continuum from gender sensitive to gender transformative [43], we categorised the studies as decolonial neutral (no decolonial issues taken into consideration); decolonial sensitive (recognises decoloniality issues but does not address them); decolonial responsive (recognises issues and applies methods to change the situation); and decolonial transformative (recognises decoloniality and applies methods and mechanisms led by Indigenous peoples) and explored the relationship with stewardship of the process (who leads or takes the initiative).

## 3 Results

The following sub-section presents the results for the first two review questions and characterises the knowledge integration studies and the knowledge types they aim to integrate. Next, we move to the methods and procedures used to integrate knowledge. Then, we assess the inclusivity of the methods, focusing on the degree of collaboration. The last two sub-sections address the opportunities and challenges of knowledge integration and co-production in the reviewed projects and the extent to which the debate on decolonising knowledge plays a role in the initiatives.

### 3.1 The evidence base: knowledge integration projects in Southern Africa relevant for conservation and NRM

From an initial set of 823 papers, 14 studies met the inclusion criteria. Despite the limited evidence base, it represents a geographic spread covering five countries: Botswana (n = 2), Namibia (n = 3), South Africa (n = 4), Zambia (n = 3), and Zimbabwe (n = 2) (Table 3). The search did not identify any relevant articles from Lesotho or Swaziland. Most articles (n = 10) are case studies in which scholars initiated a project. The four remaining papers are on NRM or conservation initiatives aiming at knowledge integration [[44], [45], [46], [47]]. Table 3 presents an overview of the scope of the reviewed studies.

**Table 3:** Overview of the reviewed studies.

Reference	Country	Scope of initiative	Indigenous and local knowledge dimension	Other knowledges systems
Buthelezi et al., 2013 [48]	South Africa	Create new knowledge by integrating knowledges for land evaluation and soil fertility studies; influence policy.	Conservation methods; livelihood traditions (crop manure)	Empirical knowledge obtained through interviews and soil analysis
Costant & Taylor, 2020 [49]	South Africa	Create new knowledge: ILK can inform an ecosystem services framework; influence policy.	Livelihood traditions (food security); taboos and beliefs; sacred places; climate indicators	Empirical knowledge obtained through participant observation and semi-structured interviews
Del Rio et al., 2018 [50]	Zambia	Create new knowledge by integrating local knowledge and remote sensing for eco-type classification; influence policy.	Conservation methods	Empirical knowledge obtained through focus group discussions, participatory mapping, remote sensing
Eisold et al., 2006 [51]	Namibia	Create new knowledge by integrating, comparing and synthesising anthropological and ecological data on essential elements of pastoralist range management; influence policy by generating new insights for decision-making processes.	Conservation methods	Empirical knowledge obtained through free listing and vegetation analysis
Jevon and Shackleton, 2015 [52]	South Africa	Create new knowledge by integrating information from elderly respondents with standard ecological surveys.	Conservation methods	Empirical knowledge obtained through interviews and aerial photography
Kasali, 2011 [53]	Zambia	Create new knowledge by integrating Indigenous and scientific knowledge systems for climate change adaptation.	Climate indicators	Empirical knowledge obtained through semi-structured interviews, focus group discussions, and historical data
Kaschula et al., 2005 [54]	South Africa	Create new knowledge by integrating local or Indigenous knowledge for coppice harvesting species within a community-based natural resource management (CBNRM) approach; empower local voices.	Conservation methods; climate indicators	Empirical knowledge obtained through interviews, focus group discussions, and statistical analysis
Nezomba et al., 2017 [55]	Zimbabwe	Create new knowledge by integrating farmers' local indicators and scientific parameters to develop criteria for assessing soil degradation on croplands.	Conservation methods	Empirical knowledge from vegetation analysis, focus group discussion, and interviews
Phuthego and Chanda, 2004 [45]	Botswana	Create new knowledge by integrating traditional ecological knowledge in CBNRM; empower local voices.	Conservation methods; climate indicators; livelihood traditions	Empirical knowledge obtained through vegetation analysis, surveys, interviews, focus group discussion
Reed et al., 2008 [56]	Botswana	Create new knowledge by integrating pastoralist indicators and ecological methods that can contribute to creating indicators that are accessible to a range of users to monitor and enhance land management and sustainability; empowering local voices.	Conservation methods; livelihood traditions	Empirical knowledge obtained through participatory identification and statistical analysis
Schick et al., 2018 [46]	Namibia	Create new knowledge by integrating local actors using the adaptive MAnagement of vulnerability and RISks at Conservation sites (MARISCO) method to provide an analysis of the socioenvironmental conditions; empower local voices; address power relations.	Conservation methods	Empirical knowledge obtained through the MARESCO method, statistical analysis, and satellite maps
Sichula et al., 2016 [57]	Zambia	Create new knowledge by integrating knowledges into governmental water and sanitation programmes.	Livelihood traditions	Practitioners' knowledge for a water and sanitation programme; empirical knowledge obtained through focus group discussions, literature review, and in-depth interviews
Sola, 2005 [47]	Zimbabwe	Create new knowledge by integrating grass management; influence policy.	Conservation methods; livelihood traditions;	Empirical knowledge obtained through vegetation analysis and survey
Verlinden and Dayot, 2005 [44]	Namibia	Create new knowledge by comparing Indigenous and conventional classification of environmental land units for NRM.	Conservation methods	Empirical knowledge obtained through vegetation analysis

The overview shows that most initiatives are research projects; only a few studies document NRM or conservation projects. Most projects seek to integrate local practices and scientific knowledge [[44], [45], [46], [47], [48], [50], [51], [52], [55], [56], [57]]. One paper discusses a project integrating local practices with practitioner knowledge in governmental water and sanitation programmes [57]. Most projects aim to create new knowledge while five papers seek to influence policymaking processes [47,48,50,51,49]; three aim to empower voices (e.g., those of local communities, farmers, and communities representatives) [45,46,54], and one paper aims to identify and report power relations [46] (Table 3).

The main local ILK knowledge dimensions include conservation methods, livelihood traditions, taboos and beliefs, and sacred places. Most studies investigated ILK and practices related to conservation methods, with a focus on land-use and vegetation-cover classification [44,50], water and sanitation management [57], ecosystem conservation, wildlife management, veld products utilisation and management [45], soil assessment and crop rotation [48], and climate indicators [53].

Livelihood traditions are also explored when there is a need to understand activities and knowledge that secure basic needs through sustainable resource use, such as grazing and cattle management [56] and seasonal hunting [45]. Some studies focusing on conservation methods and livelihood traditions also pay attention to local indicators for weather forecasting [45,54].

### 3.2 Procedures and methods used in the knowledge integration initiatives

All studies included in the review used mixed methods to integrate different knowledge systems, particularly ILK and scientific knowledge. Qualitative methods employed in the reviewed studies included semi-structured interviews, surveys, participatory mapping, and focus group discussions. One study [46] used the so-called MARISCO method, which entails adaptive MAnagement of vulnerability and RISks in COnservation sites. Eisold et al. (2006) [51] employed free listing, meaning people were asked to list and sort salient fodder plants.

Quantitative methods used were vegetation and soil analysis and land classification, while in some cases, geospatial methods were used, applying aerial photography and remote sensing. The data collected through quantitative methods was used to compare and triangulate the results obtained through qualitative data collection.

Table 4 reports the different ways of integrating and combining the different knowledge systems. It reveals that most studies combine data from ILK with scientific ecological knowledge, using mixed methods to collect data and evaluate the integration process positively or mixed. One study highlights the difficulty of combining the two knowledge systems for plant management, especially when the ecological importance and local salience differ in identifying the most important species [51]. Another study suggests that integration might be complicated when accurate data is lacking and make comparison impossible. However, the study identified an alternative to scientific know-how [54].

**Table 4:** Methods and integration processes used in the initiatives and their results.

**Study**	**Data collection methods**	Integration process	**Evaluation of integration**	Output		
Buthelezi et al., 2013 [48]	Interviews; soil analysis	Information on land fertility from farmers' and researchers' assessments was combined.	*Positive*: There are similarities between the two knowledge systems.	Integrated indicators for soil fertility		
Constant and Taylor, 2020 [49]	Participant observation; semi-structured interviews	Comparative analysis to explore local perceptions of the diversity of forest ecosystem services using common international classification of ecosystem services (CICES).	*Positive*: Considers the importance of integrating stakeholder values to inform deliberative decision-making.	Informative (the paper suggests considering Indigenous local knowledge to be integrated into CICES)		
Del Rio et al., 2018 [50]	Focus group discussions; participatory mapping; remote sensing	Information on land use from farmers and spatial tools was combined.	*Positive*: The eco-type map could guide agriculture research in eco-types with low conservation value, conservation efforts and research on habitat for aquatic and forest-dependent species.	Integrated indicators for eco-type classification map		
Eisold et al., 2006 [51]	Free listing; vegetation analysis	Information on plants species from farmers' and researchers' assessments was combined by 1) Compiling local and ecological inventories of fodder plants, 2) Investigating their local salience and ecological performance, and 3) Comparing local and ecological ratings of fodder plant species to identify parallels and congruencies.	*Mixed:* Local and scientific knowledge do not coincide in plant species classification. However, the study helped identify local preferences for plant species amongst pastoralists, which scientific knowledge failed to do.	Integrated indicators for plant species management		
Jevon and Schakleton, 2015 [52]	Interviews; aerial photography	Information on plant species *Lantana camara* from farmers and ecological assessments was combined.	*Positive.* The social and ecological approaches have shown a large degree of congruence in understanding the impacts of the *Lantana camara* on recruiting forest tree species in the area.	Integrated indicators for plant species assessment		
Kasali, 2011 [53]	Semi-structured interviews; focus group discussions; historical data	Compares local weather indicators with meteorological indicators.	*Positive:* Information on weather forecasting from local indicators is more accurate than meteorological indicators.	Integrated indicators for weather forecasting		
Kaschula et al., 2005 [54]	Interviews; focus group discussions	Information on harvesting habits, plant regeneration, soil properties from farmers and biological data on coppice harvesting response are combined.	*Mixed:* Local and scientific data were hard to compare due to the lack of biological data on the coppice.	Merely informative due to a shortage of available data on the post-harvest coppice response of Indigenous savanna fuelwood species		
Nezomba et al., 2017 [55]	Vegetation analysis; focus group discussions; interviews	Information on criteria for assessing soil degradation on croplands from farmers' local indicators and scientific diagnostic parameters (laboratory studies) were combined	*Positive:* A soil degradation assessment scheme – based on both knowledges – is proposed to guide rehabilitation strategies for smallholders in Zimbabwe and similar agro-ecologies in Southern Africa.	Integrated indicators for assessing soil degradation		
Phuthego and Chanda, 2004 [45]	Vegetation analysis; survey; interviews; focus group discussions	The community-based NRM project integrated information on local environmental indicators of seasons and veld products and utilisation management, wild animal species and hunting, and local land-use management and planning indicators.	*Mixed*: Traditional ecological knowledge is key in the community-based NRM project. However, several barriers exist against integrating local knowledge, such as formal education, a new political orientation (democracy), and Christianity.	Integrated indicators for veld products and wildlife management		
Reed et al., 2008 [56]	Participatory identification	The information on indicators combines pastoralists' data on land and grass management with ecological and soil-sampling methods. Specifically, indicators were identified among local pastoralists and from the literature and evaluated qualitatively by pastoralists against the criteria they developed. The indicators emerging from this process were evaluated quantitatively using ecological and soil-based methods. Information on indicators for assessing the sustainability of land management by pastoralists was combined with ecological and soil-sampling methods.	*Mixed*: The study shows how participatory and ecological methods can contribute to valid integrated indicators used by local users for monitoring sustainable land management strategies. Unfortunately, pastoralist local knowledge is poorly spread.	Integrated indicators for environmental sustainability		
Schick et al., 2018 [46]	MARISCO methods, adaptive MAnagement of vulnerability and RISks at Conservation sites; statistical analysis; satellite maps	The MARISCO approach and satellite maps enable practitioners to systematically document knowledge related to biodiversity, threats, drivers of change, and the (previous) conservation management method for a given site.	*Positive*: Integrating knowledge provides valuable information to develop robust socioecological indicators.	Informative (the paper does not specify the concrete outcomes of the knowledge integration process)		
Sichula et al., 2016 [57]	Focus group discussion; Literature review; In-depth interviews	Steps in a three-stage process towards integrating Indigenous knowledge in education for sustainable development are 1) identification of available local knowledge, 2) Isolation of local knowledge based on the collaborative selection of local knowledge relevant to a given water and sanitation project, and 3) situating and integrating local knowledge in the context of the programme.	*Positive*: There is potential for integrating local knowledge into the water and sanitation programme.	Informative (the paper does not specify the concrete outcomes of the knowledge integration process)		
Sola, 2005 [47]	Vegetation analysis; Survey	Local knowledge integration in an NRM plan was done through the participatory development of resource management strategies by promoting best practices and mitigating negative impacts on resources and livelihoods.	*Positive*: Indigenous knowledge can be important in achieving social responsibility in any development and conservation intervention.	Informative (the paper does not specify the concrete outcomes of the knowledge integration process)		
Verlinden and Dayot, 2005 [44]	Semi-structured interviews; vegetation analysis	Information on land-use units by Indigenous communities was compared with a conventional vegetation analysis to improve scientists' understanding.	*Mixed*: Indigenous environmental knowledge has several advantages and disadvantages to consider during the knowledge integration process.	Integrated indicators for land unit classification in NRM		

Indeed, most of the reviewed integration processes in the initiatives resulted in a positive evaluation of complementary knowledge, and they clearly display the benefits of such integration [[46], [47], [48], [50], [52], [55], [57],^49^,^53^]. The combined and complementary knowledge was used to develop integrated indicators for weather forecasting or climate change adaptation [53], land management [44,50,56], plant species management [52,51], soil management [48,55], natural resource and ecosystem services management [45,49], and grass and wildlife management [56]. However, besides reporting a positive evaluation of such processes, some papers also show the difficulties and obstacles that two knowledge systems face when sharing information [32]. Two studies showed that both knowledge systems were hard to combine [51,54]; Phuthego and Chanda (2004) [45] highlight how political and cultural dimensions contribute to eroding ILK and practices, and Reed et al. (2008) [56] show that some pastoral knowledge is poorly used. This was mainly because the two systems consider different information relevant, so they focus on different empirical data and information. In several studies, the evaluation of knowledge integration was poorly explained and discussed; therefore, the authors considered the findings on local knowledge as merely informative, without further implications for NRM or conservation projects [46,47,57,54].

### 3.3 Participation of Indigenous and local knowledge holders: degrees of collaboration

All studies (n = 14) show a low level of collaboration between the different knowledge holders. Most use a consultative collaboration approach to collect knowledge and information from the participants [47,52,57,55,54]. Similarly, three initiatives use a setting-up collaboration procedure that guarantees a two-way discussion amongst different knowledge holders [44,45,56]. Although two specific cases display a low level of collaboration methodologically (setting-up collaboration), both ensure and provide a high level of collaboration at thedissemination stage by sharing and discussing the results of the research with the participants [[44], [56]].

### 3.4 Opportunities and challenges for knowledge integration in conservation and NRM initiatives

Opportunities for knowledge integration are fivefold (Table 5). The first relates to the additionality of different knowledge systems, whereby one fills the gaps of the other. Several studies show that ILK can be meaningfully incorporated and that both scientific know-how and ILK are more relevant for conservation and NRM when integrated [[48], [52], [57],^53^]. Some studies demonstrate that integration is a way to create integrated indicators for vegetation, soil and biodiversity assessments [[44], [45], [46],^56^]. Using local indicators for weather forecasting is also an efficient alternative – yet not fully explored – particularly when scientific knowledge is scarce [46].

**Table 5:** Opportunities and challenges of knowledge integration.

Study	Opportunities	Challenges
Buthelezi et al., 2013 [48]	Complementary; local empowerment	Not applied locally
Del Rio et al., 2018 [50]	Complementarity	Not addressed
Eisold et al., 2006 [51]	Identify local preferences	Incompatibility
Jevon and Schakleton, 2015 [52]	Complementarity	Not addressed
Kasali, 2011 [53]	Complementarity; identify a local perspective	Indigenous knowledge is not scientifically proven
Kaschula et al., 2005 [54]	Identify local preferences	Incompatibility
Nezomba et al., 2017 [55]	Complementarity; local empowerment	Not addressed
Phuthego and Chanda, 2004 [45]	Complementarity; identify local preferences	Recognition; power imbalances; erosion of local knowledge
Reed et al., 2008 [56]	Complementarity; context-specific; enhanced monitoring and evaluation process	Incompatibility
Schick et al., 2018 [46]	Complementarity; context-specific; result sharing	Power imbalances; mutual distrust; recognition
Sichula et al., 2016 [57]	Complementarity	Knowledge erosion;
Sola, 2005 [47]	Local empowerment	Knowledge integration is time-consuming; resource mobilisation
Verlinden and Dayot, 2005 [44]	Complementarity; mutual learning; context-specific	A lack of clear methods and procedures for knowledge integration

Second, integrating different knowledge systems might contribute to identifying local preferences, for instance, regarding fodder plants and their performance [51], preferences for more valid climate indicators when meteorological forecasting is not reliable [45,53], and preferences regarding harvesting techniques [56].

Third, integrating scientific and ILK helps capture relevant context-specific knowledge [44,46,56]. For instance, Schick [46] highlights how ILK is a rich source of site-specific information, whereas Verlinden and Dayot [44] stress the importance of local information for outsiders who apply and work in different landscapes with different perspectives and cultures.

Fourth, knowledge integration enhances results sharing, communication, and mutual learning [44], favouring monitoring and evaluation processes [56].

Fifth, knowledge integration can contribute to local empowerment, especially where it leads to a greater role in policy and decision-making processes and self-determination [47,48,55]. For example, as several studies show, developing robust integrated indicators or climate forecasting to manage natural resources enables the opportunity to generate evidence-based policy and involve ILK holders [44,45,55,56,53].

Nevertheless, challenges abound, too (Table 5). First, complementarity is as much a challenge for knowledge integration as it is an opportunity. Some papers reveal that the two knowledge systems do not focus on the same variables and, therefore, do not use the same kind of indicators. For instance, Kaschula et al. [54] found that, although anthropological and biological data is available, any structured comparison is impossible due to data scarcity and limited coherence of tree harvesting and regeneration data. Similarly, some papers show that local classifications of tree species mismatch data on vegetation coverage collected through ecological methods [51,56,53].

Second, even when integration achieves complementarity, as with Buthelezi et al. [48], the main challenge remains understanding land-use management strategies farmers apply locally or finding scientific evidence for them. For instance, Kasali [53] found that local weather indicators were more reliable than formal weather forecasting, but the main limitation was the lack of scientific evidence for ILK and practices.

Third, the lack of recognition and a failure to acknowledge power imbalances, especially the limited representation and participation of marginalised groups and their political rights, results in a lack of validation of ILK [42]. When ILK collides with scientific knowledge, there is a limited understanding of how the two knowledge systems differ or are complementary, and scientific knowledge outcomes and explanations usually prevail over ILK systems and holders. The latter, thus, have no authority to raise their voice against different knowledge systems and holders. As explicitly shown in one case, these issues might also be a pre-condition for mutual mistrust [46].

Fourth, research methods and approaches to knowledge integration remain a challenge. Unclear methods may negatively impact collaboration between local people and researchers, especially when computer-based models prevail in data collection and analysis [44], Finally, time seems to be both an opportunity and a limitation for knowledge integration. One study shows that when mutual trust is established, integrating ILK tends to lead to faster (and, therefore, cheaper) application [44].

### 3.5 Stewardship, degrees of collaboration and decolonial perspectives

We developed four variables to assess the decolonial perspective of the reviewed papers. The first is university affiliation to determine the stewardship of the research. Six studies were led by local scholars or research organisations [45,[48], [52], [57],^54^,^53^]. Five studies were led by researchers from a country other than the one where the research was carried out. Three papers involved both local and external scholars [44,55,49].

Second, we examined whether a local or external author led the publication. Based on institutional affiliation, nine studies have a local lead author; four have an external lead author; and one paper, written by two authors, is the joint work of an external and local researcher (Table 6).

**Table 6:** Decolonial perspectives in the reviewed studies.

Study	Lead author^a^	Decolonial perspective^b^
Buthelezi et al., 2013 [48]	Local	Decolonial neutral
Constant & Taylor 2020 [49]	Local	Decolonial neutral
Del Rio et al., 2018 [50]	External	Decolonial neutral
Eisold et al., 2006 [51]	External	Decolonial sensitive
Jevon and Schakleton, 2015 [52]	Local	Decolonial neutral
Kasali, 2011 [53]	Local	Decolonial responsive
Kaschula et al., 2005 [54]	Local	Decolonial sensitive
Nezomba et al., 2017 [55]	Local	Decolonial neutral
Phuthego and Chanda, 2004 [45]	Local	Decolonial sensitive
Reed et al., 2008 [56]	External	Decolonial neutral
Schick et al., 2018 [46]	External	Decolonial responsive
Sichula et al., 2016 [57]	Local	Decolonial neutral
Sola, 2005 [47]	Local	Decolonial neutral
Verlinden and Dayot, 2005 [44]	External/Local	Decolonial neutral

a Determined based on institutional affiliation.

b See Section 2.3 for clarification.

Third, we applied degrees of decoloniality to identify if and which actions were followed in the research process(see Section 3.5). Most studies (n = 9), either led by local or external scholars or institutions, are decolonial neutral, hence do not consider any decolonising issues [44,[47], [48], [50], [52], [55], [57],^56^,^49^]. Three papers are decolonial sensitive: they recognise decolonising issues but did not address them in the research [45,51,54]. Finally, two papers can be qualified as decolonial responsive; they recognise decolonial issues and apply methods to change the situation [46,53]. None of the included studies can be described as decolonial transformative.

The review further shows no correlation between local leadership and decolonial sensitivity, which implies that there is no link between the origin of the initiators of the initiative and the aim of the paper to address decolonial issues.

Fourth, we applied a ladder of collaboration (Fig. 3) to identify the different degrees to which scholars and local communities collaborate. Although most initiatives evaluate different knowledge integration processes positively, all projects (n = 14) show a low collaboration level between the different knowledge holders (Table 7). Most initiatives used a consultative collaboration approach to collect knowledge and information from the participants. Two studies revealed a low level of setting-up collaboration but ensured and showed a high level of collaboration at the dissemination stage by sharing and discussing the research results with the participants [44,56] Only three initiatives use a setting-up collaboration procedure that guarantees a two-way discussion amongst different knowledge holders in all project stages [44,45,56].

**Table 7:** Degrees of collaboration.

Study	Consultative collaboration	Setting-up collaboration	Dissemination collaboration
Buthelezi et al., 2013 [48]	**+**		
Constant and Taylor, 2020 [49]	**+**		
Del Rio et al., 2018 [50]	**+**		
Eisold et al., 2006 [51]	**+**		
Jevon and Schakleton, 2015 [52]	**+**		
Kasali, 2011 [53]	**+**		
Kaschula et al., 2005 [54]	**+**		
Nezomba et al., 2017 [55]	**+**		
Phuthego and Chanda, 2004 [45]		+	
Reed et al., 2008 [56]		+	+
Schick et al., 2018 [46]	**+**		
Sichula et al., 2016 [57]	**+**		
Sola, 2005 [47]	**+**		
Verlinden and Dayot, 2005 [44]		+	+

a No studies scored on ignorance, information, co-designed collaboration, or co-managed.

collaboration (c.f. Table 3), so these categories are left out of this table.

## 4 Discussion

### 4.1 Evidence of knowledge integration: the need for a critical analysis

Most literature agrees that integration is a “good thing” [[58], [59], [60], [61]]. So does our review, with results showing that knowledge integration processes can enhance collaborative processes. However, our results also show a large discrepancy between what the researchers say they find important in knowledge integration and collaboration and how they act upon it. For instance, the different degrees of collaboration demonstrate that integration occurs at an advanced stage of the research (e.g., consultative collaboration with a one-way flow of information), and collaboration with local knowledge holders is mainly an extraction of what the research needs to incorporate into scientific knowledge. Knowledge integration should involve local knowledge holders from the design phase of a project until the dissemination stage, which can be described as knowledge co-production [19].

Also, many issues initially seem to be just technical and methodological. However, a deeper, more critical analysis reveals important considerations for social and governance aspects and power relations [23,62]. For example, the evidence base highlights that efficiency (participation as a tool for better outcomes in a project), empowerment (participation as a tool for improving people's livelihood), and knowledge production are the main scopes of all initiatives. However, the efficiency outcome only refers to new scientific knowledge production, while for empowerment, there are no sufficient explanations of how local people's and community empowerment occurs, or livelihoods improve.

Several international agreements aiming to amplify the voices of the most marginalised groups in decision-making processes have perpetuated a rush for participation in environmental and development initiatives. We suggest that such endeavours must be alert to the potential of what has been described as the ‘tyranny of participation’ and ensure that their efforts are not overriding legitimate existing processes or reinforcing existing power dynamics [63]. Actions that formalise local participation are often insufficient to empower marginalised groups in decision-making processes, including in the research field [64]. Meanwhile, there still remains limited concrete evidence to demonstrate that participation effectively enhances the livelihoods of the most marginalised groups [59] and helps create more equitable forms of collaboration [60]. Without denying the relevance of integration nor disregarding the attempts we present in the review – we suggest that a need remains for a more critical analysis of participatory and collaborative approaches to understand their limitations and strengths more fully and better inform development policy debates.

### 4.2 Unclear procedures and power dynamics

Selecting appropriate methods and techniques is another key ingredient for efficient knowledge integration [59,65]. Methods and procedures are important in defining the degree and the development of knowledge integration processes. Our review suggests that knowledge integration only occurs if scientific knowledge has the instruments to validate local knowledge and practices. For instance, when local indicators clash with scientific indicators, the comparison is challenging, and, in some cases, the research shows data incompatibility as the ultimate result, with no further investigation into cultural differences and how different priorities or epistemologies might lead to such divergence of relevant approaches and outcomes. Researchers seem to perceive local culture with a double standard: on one side, it is a positive feature that provides ‘alternative’ information for data collection; on the other, it is seen as a constraint that prevents the integration of local and scientific data.

For instance, in our review, conservation methods are the local knowledge dimension most commonly combined with scientific knowledge. Many other local knowledge dimensions, especially taboos and values, are absent and potentially the most challenging to be integrated with scientific know-how. Again, some literature explains that such complex application of integrating different knowledge occurs because of the many different perspectives of what constitutes knowledge and how to integrate different knowledge systems, with specific ontological and epistemological challenges [61,66].

The most used research strategy is mixed methods, creating several options for integrating local knowledge into research initiatives [67,68]. Geospatial tools are where integration occurs the most. They are practical strategies to verify and validate information and perspectives of local knowledge holders [[69], [70], [71]]. While quantitative methods for knowledge integration validates local knowledge outcomes, qualitative methods such as surveys, semi-structured interviews, and participant observation offer the possibility to navigate perceptions and culture related to the use of knowledge [72].

Indigenous and local people can be interviewed to share their knowledge, but they can also co-lead activities in the field [4]. But it appears that this still occurs far too infrequently. Our review suggests that interviewing seems to be the most popular method to collect data on local knowledge, but co-design – a strategy never used in the cases reviewed – appears to lead towards more integrated outcomes [19]. Indeed, higher levels of engagement become a route to the empowerment of local actors who actively relate to different knowledges, beliefs, and worldviews [58]. Adequate research instruments, such as new ways of gathering data and better collaborations between local and research knowledge (e.g., visual tools), can incentivise recognition and valuation of different knowledge systems and stimulate knowledge integration. It may also help understand the cultural and ecological embeddedness of Indigenous or local ways of knowing [73,74]. Such inclusive data collection methods include historical trend analyses, scenario building, participatory mapping, participatory GIS tools, and photovoice activities [50,69,75].

To avoid marginalising knowledge and properly value ILK, stewardship and co-management are considered essential steps towards knowledge integration [76]. Therefore, documenting local knowledge is an important preliminary step towards sharing and integrating knowledge with a broader range of tools while allowing adaptation to local realities. Although local knowledge documentation has been one of the pillars of engagement with other knowledge systems, literature reviews alone are insufficient [77]. Identifying participatory approaches that make local knowledge documentation more collaborative is necessary. A case in point is MacLeod's [78] recent paper, which suggests creating a citation template for Indigenous elders and knowledge keepers to acknowledge Indigenous voices within academia. Stewardship and co-management in both academia and governmental institutions need to acknowledge ILK experience and also recognise non-written forms of knowledge [8,27]. Participation and integration can also be “tyrannical” by ignoring the political differences between stakeholders, interests, worldviews, and cultures [63]. Hence, valuing and validating local knowledge means addressing the intrinsic dynamics of power sharing and imbalances.

By going beyond integrating knowledge and engaging with various knowledge systems at various degrees, a ‘political forest’ of discourses, knowledges, governance, and power issues emerges [79]. Scholars often recognise that asymmetric power issues arise when connecting science with locally based knowledge systems [80]. Similarly, an increasing number of studies recognise the importance of managing power dynamics and addressing the tendency to depoliticise such integrated processes [8,14,30,58,81].

Recognition of power relations is a step in the right direction, albeit insufficient. Attempts at integrating different knowledge systems must uncover and address the multiple ways power is held and exercised, including navigating both visible forms of power, such as the power of the private sector, and hidden manifestations of power (manipulating agendas by gifts and donations), to help understand how knowledge and its production shape social relations [82].

### 4.3 Exploring the politics of knowledge through a decolonial approach

Exploring the politics of knowledge in a landscape requires, first, establishing an evidence-based discourse of sustainable development as a co-produced pluralistic debate on politics, knowledge, and claims [8,58,83]. Second, as expressed in post-colonial theories, there is a need to go beyond modernity through decolonised perspectives [[84], [85], [86], [87], [88], [89]]. This implies going beyond ‘incorporating’ and ‘including’ ILK into conservation and NRM research. Decolonising processes should be ‘two-eye seeing’ practices, which means co-development in every step of the research project or process from design to implementation and dissemination [19,24], as well as knowing histories and philosophy of science [90].

Development and conservation processes in the Global South have often been guided from a colonial perspective. As such, participatory action research and multi-stakeholder platforms are increasingly considered appropriate strategies to engage local voices actively and achieve more inclusive and collaborative processes [58]. Given this, it was somewhat surprising that our review found that scholars or organisations, despite using various integration methods and degrees of collaboration, typically failed to explicitly recognise or address decolonial issues. For instance, few papers openly address decolonial issues by using proper methods that facilitate the use of local knowledge within the project, while some papers do not recognise decoloniality at all and fall into the ‘incompatibility trap’ between scientific know-how and local knowledge. Others who recognise decoloniality issues briefly mention it in the conclusion section as a need for further research. None of the reviewed papers activated a transformative approach in the implementation phase that addresses the politics behind knowledge integration processes.

Moreover, many cases show that integration processes revolve around the idea of inclusivity without establishing concrete action to achieve it [46,47,57,49,54]. Worryingly, local knowledge and knowledge holders were ‘inadequately’ considered before or after the research process, and the reviewed cases failed to explain how such processes developed. Hence, the need to focus not only on collaborative methods and procedures but also on how such approaches are being developed, with whom, and why. Such deeper exploration might help address social and power dynamics that interfere with and shape decision-making processes [8,19,44,45,48,55,50,58,91].

Our review shows no correlation between local authorship and decolonial issues. Therefore, it can be argued that recent attempts at integration often maintain neo-colonial practices. However, regarding authorship, the results suggest something different, with the majority of the reviewed studies being led by local researchers working in Global South research institutes. While this is an encouraging result, patterns from the broader literature suggest this cannot be generalised and much still needs to be done. In 2003, a study revealed that despite researchers claiming to carry out collaborative research with institutes in the Global South, 70 % of the published research failed to acknowledge the contribution of Global South researchers in co-authorship [92]. A recent plant sciences review [93] revealed multiple biases that continue to influence publishing within this field, particularly gender and geography (correlated with affluence).

Similarly, Smith et al. [94] found that scholars from non-English and low Human Development Index (HDI) countries were significantly disadvantaged in the peer-review and publication process. There are several reasons, including colonial legacy, limited funding, and the difficulty for scholars from the Global South to meet Western standards for academic publishing [95]. However, a major contributing factor is the use of English as the *lingua franca* for international science. Since a call in Current Contents in 1967^6^ to publish as much as possible in English, it continues to dominate journal publication systems until today.^7^ As such, it often acts as a gatekeeper of scientific discourse [96]. For instance, 80 % of articles in the Scopus index are published in English, and journals previously publishing articles in other official languages (e.g., Animal Biodiversity and Conservation in Spain, Natureza & Conservação in Brazil) have decreased publishing in these languages [96]. By ignoring other languages than English, there is the risk of provoking challenges and gaps in the transfer of knowledge [96].

For over twenty years, the scientific community has expressed concern about the lack of linguistic diversity, primarily because of the implications for science of the exclusion of relevant knowledge and biases [97] Although not all Southern scholars are aware of or concerned about the issue, a growing number are making a strong case for decolonising science based on their lived experiences with language and publication barriers [[95], [96], [97]]. Therefore, we argue that expanding formal and informal science communication beyond the English language can make science more accessible and participatory [97,98]. Although a common language is necessary for scientific communication, developing multilingual alternatives would promote diversity [99].^8^

In this regard, decolonising knowledge (both as object and medium) could offer a way forward towards addressing power relations and integration by exposing places and dynamics where dominant structures act to change such dynamics into new equitable ways of co-creating knowledge [23,62]. Some authors have suggested that to transition to decolonisation, epistemic disobedience is first required [100] to trigger the chance to rethink landscapes as visual sceneries, abstract ecologies, autonomous cultural spaces, interests, and conflicts [101]. Decolonising action should therefore start prior to integration attempts, whereby before reconciling differences, there is a need to recognise and address those irreconcilable relics from colonial structures and legacies [101,102] are still often considered an ‘alternative or radical way’ to manage natural resources.

Creating knowledge integration and co-production strategies is an important step but likely insufficient without considering the broader political and social context. In the international arena, the 2021 United Nations climate change conference (COP26) recognised the contribution of IPLCs in forest protection by pledging 1.7 billion dollars to these communities [19,24,101,[103], [104], [105]].

### 4.4 Why integrating local knowledge is important

Despite enthusiasm for pursuing knowledge integration processes, knowledge gaps for integration are twofold: the limited empirical evidence and the lack of documented cases of negative impacts of knowledge integration in sub-Saharan Africa. As mentioned above, a body of literature questions the feasibility of integration due to ontological and epistemological differences between local and scientific knowledge.^9^ If this is the case, we would expect our review to show some negative outcomes or at least highlight the inherent challenges better, but this is not the case and hints at potential research or publication bias.

Research has demonstrated that the forest loss rate is significantly lower within Indigenous people's land [97], yet Indigenous people's rights within such lands are often still not recognised. Establishing and respecting clear land tenure rights would enable IPLCs to play an active role in NRM decision-making processes concerning conservation and landscape governance [24,85,86]. Although this was widely – and rightly – applauded, how much of this commitment actually reaches IPLCs remains to be seen. 10.13039/100014337Furthermore, there was little consideration of the impact of such a volume of funding reaching IPLCs. Would this push IPLCs towards market economies, and what would this impact be for IPLCs and their lands?

Moreover, are local capacity and external support (or both) in place to facilitate the structural and institutional changes required to sustainably secure and improve IPLCs' land, livelihoods, and well-being? Such changes will require a greater focus on colonial legacies, IPLC rights, and questioning the assumptions underlying the call for greater integration of local and scientific knowledge. Our review suggests that, thus far, this is happening infrequently and superficially, and further and deeper examination of integration processes is required to understand how and under what conditions it can be (reciprocally) effective. It is time to go beyond the ‘seat at the table’ rhetoric and reconstruct assumptions behind integrating IPLCs' knowledge.

### 4.5 Limitations of the review

This review has several limitations, notably concerning the limited number of case studies and its geographic focus on Southern Africa. Hence, this review cannot fully represent the reality behind knowledge integration processes regarding research projects for conservation and NRM at broader scales or other geographies. Moreover, the selection of search terms used to retrieve relevant publications was challenging due to the multiple and often confusing or vague ways in which knowledge integration is described. We recognise the complexities and sensitivities associated with certain terminology. For example, knowledge integration can be co-opted and rather reflect assimilation, but for the purposes of this review and to capture a sufficient breadth of literature, we used the term knowledge integration, which many papers still use to refer to knowledge co-production and co-creation. Hence, relevant publications may have slipped our attention. We tried to compensate for this through additional hand-searching in relevant journals and reference lists. Moreover, we realised that the inconsistent terminology and the language used in the reviewed studies make interpretation, analysis, and comparison challenging.

Despite these limitations, our review and reading of the broader literature suggest that knowledge collaboration and integration strategies remain largely undocumented or poorly explained, limiting our understanding of patterns or guidelines across the initiatives. Hence, these findings present a starting point and a strong case for a more critical analysis of methods and approaches in initiatives aiming to integrate different knowledge systems at the practical and policy level.

## 5 Conclusion and the way forward

Balancing and respecting different knowledge systems is pivotal to enhancing decision-making processes in conservation and NRM research and projects. Yet, this review shows that integrating ILK and scientific knowledge (or other knowledge systems) remains nascent. The literature on knowledge integration is evolving. Whether called knowledge integration or knowledge co-production, such processes should aim for a transformational change, but there are still many limitations to achieving that. Indeed, achieving transformational change first requires establishing transformative processes. A shift from integration to genuine collaboration across different knowledge systems requires meaningful participation of knowledge holders from design to dissemination, addressing power imbalances, and a commitment to decolonising conservation and NRM. Rethinking and reshaping the politics of knowledge is essential for more integrated and collaborative conservation and NRM processes.

Despite the growing emphasis on integration and co-production approaches, knowledge integration remains asymmetrical, as conservation and NRM decision-making processes largely occur without considering equitable spaces for context-embedded knowledge of local realities. Moreover, documentation of procedures that ensure integration and empower local knowledge holders is still scattered and fragmented. Issues of power imbalances and decolonising knowledge are partially and superficially addressed in the reviewed studies. Similarly, opportunities remain unexploited, and challenges are overlooked.

In conclusion, we argue that although the results are specific to Southern Africa, they hold relevance for other tropical contexts where the potential to better integrate local and scientific knowledge exists. There is a pressing need for further research on knowledge integration at the practice and policy levels and to move beyond knowledge sharing and mutual learning towards decolonising knowledge for conservation and NRM. Moreover, more research is needed on methods and strategies for knowledge integration. The politics of knowledge in shaping stakeholders’ interactions, power relations, and dialogue amongst different knowledge systems deserve more attention. Finally, we call for research and policy that stimulate a critical rethinking of what knowledge integration means and to whom and how it can positively impact the most marginalised groups and the interactions of different knowledge systems and holders.

## Data availability

Data will be made available on request.

## CRediT authorship contribution statement

**Malaika P. Yanou:** Conceptualization, Data curation, Formal analysis, Investigation, Methodology, Writing – original draft, Writing – review & editing. **Mirjam Ros-Tonen:** Formal analysis, Methodology, Supervision, Writing – review & editing. **James Reed:** Funding acquisition, Supervision, Writing – review & editing. **Kaala Moombe:** Validation. **Terry Sunderland:** Funding acquisition, Supervision, Validation, Writing – review & editing.

## Declaration of competing interest

The authors declare that they have no known competing financial interests or personal relationships that could have appeared to influence the work reported in this paper.

## Footnotes

1 In this paper Indigenous and local knowledge (ILK) “refers to the understandings, skills and philosophies developed by societies with long histories of interaction with their natural surroundings. For rural and Indigenous peoples, local knowledge informs decision-making about fundamental aspects of day-to-day life.” [34]. We use this term throughout the paper unless we cite from reviewed papers that use another term.

2 This study is part of a broader study on the role of Indigenous and local knowledge in the context of integrated landscape approaches with a focus on Zambia [106,107]. Therefore, we decided to focus this review on Southern Africa, as other contexts might be too different to draw meaningful conclusions relevant to the overall study. However, we acknowledge that the general findings regarding the lack of documented evidence of knowledge integration process and a lack of commitment to decolonial issues have broader implications (see the concluding section).

3 In this paper, we consider decolonising knowledge as a way to go beyond “incorporating” and “including” Indigenous and local knowledge into conservation and NRM research. We believe a decolonising process implies ‘two-eye seeing’ practices, which means co-development in every step of the research project or process from design to implementation and dissemination [19,24].

4 After several attempts in all the databases, we decided to exclude the terms related to “knowledge integration” OR “knowledge co-production” OR “knowledge co-creation”, as the number of hits provided was insufficient for any meaningful analysis. For the search, we used TITLE + ABS + KEYWORDS for Scopus and searched by topic in the Web of Science.

5 Environmental Science and Policy, Human Ecology, Sustainability Science, and Environmental Management.

6 See http://garfield.library.upenn.edu/essays/V1p019y1962-73.pdf.

7 English has not always been the *lingua franca* of science; it has only been so since the last half of the last century Previously, Latin, French, and German were more common in the Western world, with Esperanto promoted in vain [96,97,108].

8 Managers of scientific journals, however, would claim that this is not efficient and cost-effective and therefore unfeasible. See, for instance, Ref. [109].

9 Following Marsh et al. [111, p. 178], we understand ontology as “the form and nature of reality” and epistemology – the “theory of knowledge” – as the ideas about how we can get to know the world.
